# TTR-FAP: liver transplant vs oral medication. How and when

**DOI:** 10.1186/1750-1172-10-S1-I6

**Published:** 2015-11-02

**Authors:** Teresa Coelho

**Affiliations:** 1 Unidade Corino de Andrade, Centro Hospitalar do Porto, Portugal

## 

Liver transplant was introduced as a treatment for Familial Amyloid Polyneuropathy (FAP) in 1990 and since then it was demonstrated that the best results are seen with early onset patients with predominant neurologic involvement (typically Val30Met patients), at the first years of symptomatic disease. Complete stabilization of the neuropathy is seen in a significant proportion of patients. However the treatment introduced mortality and morbidity due to the surgical procedure itself, variable from center to center.

Tafamidis, a transthyretin (TTR) stabilizer, administered orally, was approved in Europe in 2011 to treat neurologic involvement of stage 1 patients with any TTR mutation. We have limited long term data on the efficacy of this treatment and after starting treatment we need at least 6 to 12 months to evaluate the impact of the drug on disease progression. This means that choosing Tafamidis first, may significantly delay the option for liver transplant, in case of oral treatment failure, impacting the results of surgery. The choice between these options is not easy because we have not yet enough comparable data.

Most patients would prefer to avoid or to delay the need for an aggressive treatment, hoping to be part of the group of good responders to oral medication. How much should doctors point to a given option?

Oral treatment should be prescribed as soon as patients develop the first symptoms and signs of neuropathy. Patients diagnosed with advanced stage 1 disease with high probability of being refused for liver transplant in one year should be sent directly to surgery. If the waiting time is less than one year the benefit of receiving Tafamidis while waiting must be weighted.

Patients with more advanced neurologic disease and/or important cardiac involvement should be considered for clinical trials or for other drug treatments.

